# Supplementary material for: MetaRibo-Seq measures translation in microbiomes
Source: Nat Commun. 2020 Jun 29;11:3268. doi: 10.1038/s41467-020-17081-z (PMC7324362; doi:10.1038/s41467-020-17081-z)
Supplement: Supplementary file 10 — Supplementary Data 7 [file 41467_2020_17081_MOESM10_ESM.zip › File2/Confidence_VeryHigh_Taxonomy/224654_out.krona.html]

Javascript must be enabled to view this page.

members
magnitude
magnitudeUnassigned
count
unassigned
taxon
rank

224654\_out

9

9
2
superkingdom

phylum
9
1239

class
9
186801

186802
9
order

family
9
541000

9
216851
genus

9
853

SRS015663\_contig\_number\_36022SRS049896\_contig\_number\_2311SRS076804\_contig\_number\_21021SRS097920\_contig\_number\_contig-100\_2005.40368SRS1041038\_contig\_number\_7251SRS1041136\_contig\_number\_20330SRS104311\_contig\_number\_688SRS147653\_contig\_number\_3493SRS148091\_contig\_number\_11308
species
